# Securing continuation of treatment for children with cancer in times of social unrest and pandemic

**DOI:** 10.1002/cnr2.1430

**Published:** 2021-05-31

**Authors:** Verónica Zuleta, Josefina Berliner, Nuria Rossell, Marcela Zubieta

**Affiliations:** ^1^ Fundación Nuestros Hijos Santiago Chile

**Keywords:** childhood cancer, Chile, Covid‐19 pandemic

## Abstract

**Background:**

Childhood cancer in Chile reports 500 new cases each year of which 85% are treated in the public health system. Governmental programs ensure access to diagnosis, treatment, follow up and palliative care, whereas Fundación Nuestros Hijos (FNH) provides supportive care for non‐covered medical and psychosocial needs. Common financial difficulties in families of children and adolescents with cancer increased considerably when a wave of social unrest arose in October 2019 and the Covid‐19 pandemic in March 2020 hit the country, leaving families of children with cancer facing greater challenges.

**Aims:**

We report here the support activities and interventions carried out by FNH to help the families during the crisis of these months.

**Methods:**

A socioeconomic survey was conducted among FNH's beneficiary families to know their needs. During these months of acute crisis for many families, support activities and interventions were developed and varied types of aid were allocated to help the families.

**Results:**

The main results of the survey in which 525 (70%) of FNH's beneficiary families participated showed that 75% of them had only one breadwinner, and 52% had one unemployed family member. Almost 90% of job loss happened during the months of social unrest and pandemic. Four main interventions: (a) safe transportation, (b) food, (c) heating, (d) internet connectivity, were organized to support important needs of the families and prevent children to miss treatment appointments. Additionally, some families who did not access governmental emergency aid were guided in the process.

**Conclusions:**

The aid provided helped the families to relieve some of their needs, facilitated the continuation of treatment during the pandemic, and made the caregivers feel supported and listened.

## 1 INTRODUCTION

With about 500 children under 15 years diagnosed with cancer annually, and an estimated 1000 children and teenagers in treatment each year, Chile has a childhood cancer survival rate of 78%.^1^, ^2^ About 85% of all diagnosed children are treated in the public health system through the Pediatric Antineoplastic Drug Program^3^ which offers comprehensive services including diagnosis, treatment, follow‐up, and palliative care. Access and financial support are secured through the Explicit Health Guarantee Program,^4^ which provides a set of guaranteed‐by‐law benefits regarding access, quality, opportunity, and financial protection for an ample number of chronic and life‐threatening diseases, including childhood cancer and palliative care.

It has been widely documented that social inequalities affect cancer diagnosis, treatment access or adherence, and clinical outcomes.^5^ This is the case even in countries where treatment is available free of cost since the burden of cancer treatment and poverty involves a variety of social and emotional aspects beyond financial.^6^ Living conditions determined by socioeconomic limitations, such as lack of good nutrition, access to electricity and running water, access to transportation, or housing conditions also affect treatment outcomes.^7^ This is why, to secure treatment continuation and best treatment outcomes, financial and psychosocial support is essential in these settings, where civil society initiatives represent one of the most efficient resources contributing to better results in childhood cancer.^8^

Fundación Nuestros Hijos (FNH) (Foundation Our Children), in partnership with the Chilean state, works to cover the multiple needs that arise in the course of treatment and follow‐up, to help improve the children's quality of life and survival rates. Through a coordinated set of programs that include medical services (rehabilitation and palliative care), social services, in‐hospital schools, and housing, FNH provides multidisciplinary assistance to cover or improve the benefits that the families receive through the national health system. In 2019, FNH granted 95 618 aids to 750 children and adolescents. Table 1 shows the general scope of the programs that FNH has in place to provide free of charge assistance to all children diagnosed with cancer in Chile.

**TABLE 1:** Programs of Fundación Nuestros Hijos for the improvement of childhood cancer care in Chile

Program	Coverage
Medical services	Delivering of complementary food, medicines, medical supplies, loans of medical equipment, financing of medical tests, and provision of orthoses
Social services	Financial support is granted for medical mobilization, housing improvement, and mortuary fees
Housing	With capacity to host 20 patients along with their caregiver in three guest houses in the city of Santiago during any given length of time
Rehabilitation	Comprehensive oncology rehabilitation is given through the clinical specialties of physiatry, kinesiology, occupational therapy, neuropsychology, psychology, speech therapy, dentistry, and special education
Education	Intra‐hospital schools in two health centers of the PINDA Network for the children's continuation of schooling while in the hospital, as well as support of their reintegration into their schools once discharged from the hospital
Palliative care	Comprehensive hospital and domiciliary nursing care and rehabilitation services allowing for a dignified death without pain

According to the governmental classification of household income, and FNH's 2019 records 44% of its beneficiaries were in category A, which comprises people without income, and 19% were in category B, with income below the Chilean minimum wage (USD $411). These two categories are covered by the public health system and receive treatment 100% free of charge; families in category C pay 10%, and those in category D pay 20% of their treatment costs.^9^ Although FNH provides its services free of charge to all families, this information offers a view of the level of the social and financial condition in which the families find themselves at the moment of their child's diagnosis. In line with this, in 2017, Zubieta et al.^10^ found that the families of children with cancer undergoing treatment in one of the main hospitals in Chile were dealing with limitations regarding their educational level, housing conditions, employment, and income, which could affect treatment outcomes.

Over the past year and a half, two social situations in Chile exacerbated the vulnerability of families of children and adolescents with cancer: in October 2019, a wave of social unrest started, which for several months kept the country in serious instability with outbreaks of violence that generated destruction of public spaces and infrastructure, and loss of jobs especially due to the closure of small and medium‐sized businesses. With the situation still unstable, the first contagions of COVID‐19 were reported at the beginning of March 2020. A state of constitutional emergency of catastrophe was declared, imposing limitations such as movement restrictions, lockdown, shut down of commercial activity, suspension of school attendance, etc. The Covid‐19 pandemic caused an economic growth fall close to 4% of GDP and unemployment that in June 2020 reached 9%.^11^

FNH engaged in support activities and interventions to help the families during the crisis of these months. Simultaneously, it conducted a survey to collect information about the social and economic situation of the beneficiary families, and how they were affected by these two events. The results of the survey are shown here to convey an image of the situation in which FNH deployed its efforts.

## 2 METHODOLOGY

### 2.1 Survey

A socio‐economic survey was carried out between August and September 2020 among respondents who met the following selection criteria: families of patients enrolled in the FNH registers from January 2019 to July 2020 and who had their phone numbers updated at the time of the survey.

A survey with 19 questions was developed, including closed questions with dichotomous and multiple‐choice variables, and open free‐response questions. The questionnaire included three main areas: (a) demographic characteristics, (b) employment and socio‐economic situation, (c) benefits and aid received.

A team of 19 volunteers who regularly collaborate with the foundation was formed to collect the surveys. The team was trained in the application of the questionnaire, which was conducted using the online service Google Forms. The patients' parents or caregivers were contacted by telephone and were asked their verbal consent to participate in the survey. The survey takers identified themselves as FNH representatives and explained the purpose of the survey and the confidential and voluntary nature of their participation. The answers were immediately recorded into the Google Form.

The results were tabulated. Tables of direct data and percentage summaries were done for the closed dichotomous and multiple‐selection responses. In the case of open answers, they were classified into categories.

### 2.2 Interventions

The information obtained from the survey was shared as an internal report among the several technical areas of the foundation, which kept constant group communications to coordinate, organize, and distribute the aid obtained. Not all the interventions were organized based on the survey information, but some of them were directed as much as possible towards helping problems found in the survey, especially problems that could risk the continuation of treatment, like lack of transportation or financial means to attend treatment. Four of the main support interventions are explained here: transportation, food, heating, and connectivity. An additional informal intervention regarding aid guidance is also explained.

## 3 RESULTS

Of the 745 families served by FNH between January 2019 and July 2020, 615 families met the initial criteria. Responses were obtained from 532 families who were able to be contacted by telephone. All gave their verbal consent to participate. The remaining 83 families could not be reached either because they did not answer the call or because their phones were out of order. Of the total 532 who did respond, 7 surveys were discarded for inconsistencies. Therefore, 525 (70%) of FNH's beneficiary families participated and answered the questionnaire.

### 3.1 Demographic characteristics

In 94% (494) of the total families surveyed the patient's main caregiver was a female. While the survey did not inquire about kinship, FNH records indicate that most children and adolescents with cancer are in the care of their mothers. Table 2 shows the type and size of families interviewed.

**TABLE 2:** General results of the socio‐economic questionnaire

	%	*n*
Demographic characteristics		
Nuclear families: both parents and one or more children	47	225
Extended families: formed by grandparents, uncles, cousins and other blood relatives or related living in the house	27	142
Single parent families: the child or children live with a single parent, either the mother or the father	24	126
Between 4 and 5 members	55	291
Six or more members	20.6	106
Employment and socio‐economic situation		
One or more members receiving income	88	462
Total monthly income below US$578	63	289
Families without any income	12	63
Have one unemployed family member	52	271
Suffered a wage reduction during the quarantine period	31	79
Internet access	90	472
Rely on gas for cooking and heating	84	434
Report difficulties paying basic services (water, electricity, heating, telephone) during the pandemic	39	189
Report difficulties paying housing rent and mortgage loans during the pandemic	27	130
Benefits and aid received		
Accessed financial help from the government during the pandemic	78	410

### 3.2 Employment and socio‐economic situation

While most of the families had one or more family members receiving income from work, 75% of them had only one breadwinner. About two‐thirds of the families had a total monthly income below USD $578, (USD $67 per capita for these families).

Unemployment was significant, with more than half of the families having at least one jobless member. Of the 271families who reported having an unemployed member, 40% (109) lost their job in October 2019 (the month of the social unrest starting) or earlier, and 48% (131) became unemployed between March and July 2020 when the COVID‐19 pandemic quarantine restrictions where strictest. A third of the families without unemployed members experienced a wage reduction in this period of quarantine. Forty percent of the families reported having a difficult or very difficult socio‐economic situation. The section about employment and socio‐economic situation in Table 2 shows data on reported financial difficulties of the families.

### 3.3 Benefits and aid received

About main support networks and resources, 57% (252) relied primarily on their family, and 23% (101) on foundations. Many respondents expressed gratitude for the survey call, which was valued as a moment in which they felt actively listened to, welcomed, emotionally contained, and with the trust to deliver all the information requested. The government distributed national economic relief packages, which more than three‐quarters of the surveyed families received (see Table 2). The reasons mentioned by 115 families for not receiving these aid packages were not being with their records up to date, not meeting the application requirements, not knowing how to make the applications, or being foreigners without a valid visa, among others.

#### 3.3.1 Interventions made

In the wake of the limitations that the state of emergency represented for patients and their families, it was soon evident that the proper continuation of treatment was at risk. With the slogan “Cancer does not wait,” FNH delivered aid aimed at securing access to treatment, rehabilitation, and school education. Four were the essential interventions that were incorporated or modified during the pandemic:

##### Transportation support for chemotherapy and radiotherapy treatment

FNH routinely provides transportation to treatment for children and adolescents with cancer who by medical indication should not use public transportation. Due to limited access to transport during the lockdown, and in order to avoid the risk of COVID‐19 contagion, transportation was provided to all patients to access the medical facilities safely. For the patients to reach their appointments for clinical check‐ups, chemotherapy, or radiotherapy, 1062 transportation services were delivered for a total of 180 different children and adolescents with their respective parents.

##### Food support

The survey results showed that families had difficulty paying general living expenses bills. As a way of helping to reduce food costs, a total of 682 food baskets were provided to 420 families. Also, lunch and breakfast meals were delivered to 67 parents of hospitalized patients, which meant 883 lunch meals and 407 breakfast meals. Also, 50 gift cards of US$385 each were delivered, for purchases in supermarkets.

##### Heating support

The survey showed that most families rely on gas for cooking and heating but paying for gas was an additional difficulty. To help them with some expenses, 1100 gas purchase discount tickets were delivered. Also, 258 gas stoves, 49 kerosene stoves, and 5 electric stoves were given.

##### Connectivity support

During many months of the lockdown period, the hospital schools and the rehabilitation center run by FNH had to stay closed, and an online education system was started. The same online strategy was used for telerehabilitation sessions for all children and adolescents who were enrolled in schools and those who were undergoing rehabilitation treatment. Both of these services were considered a priority for the quality of life of the children, and, although most of the families had access to an internet connection, many did not have sufficient required technology to access these services. Eighty‐two tablets, 95 phones, and 50 internet chips were delivered.

##### Guidance in government aid applications

The results from the survey showed that around 115 families did not access government aid. Although this was not an intervention systematically documented, FNH guided those families not sufficiently informed on how to access the benefits provided by the government, and in several cases, the families were helped through their application process.

## 4 DISCUSSION

The difficult socio‐economic condition of parents of children and adolescents with cancer is an ongoing challenge that represents a vulnerability for the entire family in all countries.^12^ Many studies have shown that the financial impact of having a child diagnosed with cancer is considerable and long‐lasting even in countries where treatment is subsidized or lack of finances are not an impediment to access treatment.^13^, ^14^ Out‐of‐the‐pocket expenses show to be considerably high.^15^ Additionally, such financial stressors contribute greatly to family burden and emotional distress.^16^

Due to the rigorous care that children and adolescents with cancer require, many of their parents, especially mothers must leave their work or opt for informal sources of employment, which makes their economic situation more precarious especially in emergency contexts. This is a common situation for many families of children with cancer in all countries.^12^, ^17^ The unstable employment situation of Chilean families is evidenced by the fact that 75% of the families surveyed indicate that only one member is generating an income. Family socioeconomic circumstances already at the limit while undergoing treatment for a child with cancer are easily exacerbated when a social crisis hits a nation, as we could assess with the high percentage of loss of employment (52%) among the families surveyed, mostly related to the wave of social unrest of October 2019 and the Covid‐19 pandemic since march of 2020. The increased economic precariousness of the families also increases the vulnerability of children and adolescents with cancer to both be able to continue with their treatment and to receive it in favorable terms. The collaboration of civil institutions with state programs can be of great help for families who are already too financially and emotionally overwhelmed to find their way and advocate for themselves.^12^, ^18^ This was also seen in the survey as the respondents considered the foundations to be their main support network besides their relatives and family members.

In a context of global emergency such as that of the pandemic, it is not surprising that the general population is exposed to increased vulnerability and a sense of collective unprotection. The global scale of this vulnerability, scarcity of resources, disruption of social assistance systems, etc., which affects everyone, puts at greater risk families of children and adolescents with cancer, who live in permanent vulnerability. The collectivization of vulnerability increases the risk of making the special needs of these families invisible. This coupled with the shortage or prolonged decrease in resources or funds by governments and aid foundations can represent a great burden of distress and added uncertainty. Therefore, it is valuable to highlight the gratitude expressed by the respondents of the survey, for whom the questionnaire conversation was an opportunity to feel listened, and the interventions became sources of relief.

The challenge for FNH is now to establish and/or improve mechanisms to keep updated and permanent contact with the beneficiaries, being aware and sensitive to the changing contexts and circumstances that the families face, and to the demands that occur in the different stages of the disease process. Pelletier and Bona^12^ emphasize the importance of addressing the financial burden on the families of children and adolescents with cancer as part of comprehensive psychosocial care and support. They stress the value of developing standardized measurement instruments that can facilitate evidence‐based interventions. As an opportunity from this survey experience, FNH might evaluate future initiatives to be incorporated within its standards of care, which might include evaluation and measurement instruments as well as interventions.

## CONFLICT OF INTEREST

The authors declare that there is no conflict of interest.

## AUTHOR CONTRIBUTIONS

*Conceptualization, data curation, formal analysis, methodology, project administration, writing‐original draft*, V.Z.; *Conceptualization, data curation, project administration, supervision, writing‐review & editing*, J.B.; *Conceptualization, formal analysis, supervision, writing‐review & editing*, N.R. *Conceptualization, project administration, supervision, writing‐review & editing*, M.Z.

## ETHICS STATEMENT

Respondents to our survey consented in oral form to answer the survey. For this report and analysis, all the information has been anonymized.

## Data Availability

The data analyzed for this report are available from the corresponding author on reasonable request.
